# Protective Immunity to Listeria Monocytogenes Infection Mediated by Recombinant *Listeria innocua* Harboring the *VGC* Locus

**DOI:** 10.1371/journal.pone.0035503

**Published:** 2012-04-19

**Authors:** Walid Mohamed, Shneh Sethi, Svetlin Tchatalbachev, Ayub Darji, Trinad Chakraborty

**Affiliations:** 1 Institute for Medical Microbiology, German Centre for Infection Research (DZIF), Justus-Liebig University, Giessen, Germany; 2 Centre de Recerca en Sanitat Animal, Universitat Autònoma de Barcelona, Barcelona, Spain; Federal University of Minas Gerais, Brazil

## Abstract

In this study we propose a novel bacterial vaccine strategy where non-pathogenic bacteria are complemented with traits desirable for the induction of protective immunity. To illustrate the proof of principle of this novel vaccination strategy, we use the model organism of intracellular immunity *Listeria*. We introduced a, low copy number BAC-plasmid harbouring the virulence gene cluster (*vgc*) of *L. monocytogenes* (*Lm*) into the non-pathogenic *L. innocua* (*L.inn*) strain and examined for its ability to induce protective cellular immunity. The resulting strain (*L.inn::vgc*) was attenuated for virulence *in vivo* and showed a strongly reduced host detrimental inflammatory response compared to *Lm*. Like *Lm*, *L.inn::vgc* induced the production of Type I Interferon's and protection was mediated by Listeria-specific CD8^+^ T cells. Rational vaccine design whereby avirulent strains are equipped with the capabilities to induce protection but lack detrimental inflammatory effects offer great promise towards future studies using non-pathogenic bacteria as vectors for vaccination.

## Introduction

Current state of the art vaccine technology focuses on three distinct strategies 1) the creation of live attenuated pathogens based on the deletion of virulence factors [1] 2) the use of subunit vaccines [2] which contain one or more semi-pure antigens that are critical in inducing an immune response and 3) the use of metabolically active but non-viable bacteria [3].

For the first strategy, it must be considered that in today's medicine vaccines will often be administered to immunocompromised individuals and that the use of live vaccines in such subpopulations poses serious difficulties [4], [5]. The greatest disadvantage of subunit vaccines is their general requirement for strong adjuvants, as these adjuvants often induce detrimental tissue reactions. Lastly, the concept of so-called killed but metabolically active (KBMA) bacteria involves bacteria which are unable to form colonies on growth media but still have an intact protein synthesis and secretion machinery. Such mutants are reportedly capable of inducing CD4^+^ and CD8^+^ T cell responses and protection [3]. However this requires multiple injections.

*Lm* is a facultative intracellular microorganism and many of the bacterial determinants necessary for pathogenesis, including intracellular growth and spread of *Lm*, have been identified and are clustered on a 10-kb region of the chromosome termed the virulence gene cluster (*vgc*) which encodes the *prf*A, *plc*A, *hly*, *mpl*, *act*A and *plc*B genes organized in three transcriptional units [6].

Being a facultative intracellular bacterium makes *Lm* particularly attractive as a potential live vaccine vector for the induction of cell-mediated immunity to foreign antigens [7], [8]. However, despite its capability to induce effective CD8^+^ T-cell responses the safety of recombinant *Lm* remains an important issue, as infections with *Lm* can cause severe and life-threatening infections [9]. Moreover, infection with *Lm* is mainly accompanied by undesired CD4^+^ T-cell mediated delayed type hypersensitivity (DTH) responses and granulomatous inflammation [10], [11]. Therefore the use of *Lm* in a clinical setting is associated with major risks limiting its potential as an effective vaccine vector.

An alternative strategy would entail the transfer of a core set of virulence genes from pathogenic *Lm* to create a strain that is attenuated for virulence but is capable of inducing an effective immune response. To explore this approach we have transferred the *vgc* locus of *Lm* into a non-pathogenic species of *Listeria* such as *L. innocua* (*L.inn*) as a carrier strain. Here we show that a single immunization with this recombinant strain (*L.inn::vgc*) fulfills the desired requirements for a successful bacterial vaccine vector. These include low virulence in association with induction of protective antigen-specific CD8^+^ T-cell responses and reduction of CD4^+^ T cell-mediated inflammation.

## Results

### *In vivo* survival of the recombinant *L.inn::vgc* strain

The ability of *Listeria* to survive *in vivo* at the early stage of infection is crucial for the induction of cell-mediated immunity [12], [13]. We examined the ability of *L.inn::vgc* to survive in the spleen and liver in infected mice and compared it to that of the wild type *Lm*. BALB/c mice were infected intravenously (i.v.) with sub-lethal doses of wild type *Lm* EGD-e (10^3^), *L.inn::vgc* (10^7^), or the wild type *L.inn* strain (10^7^). Time points correlating with the critical phases of host immune response to *listerial* infection were selected and used to compare bacterial growth and induction of immune effectors in wild-type *Lm*, *L.inn* and *L.inn::vgc* strains. Day 3 of a *Listeria* infection refers to the end of the pre-immune phase before the expansion of specific T cells in the mouse model of listeriosis [14]. The presence of viable bacteria on this day has been shown to be critical for the successful induction of T cell-mediated immunity [15]. Therefore on day 3, bacterial load as well as spleen morphology was analyzed. Day 9 corresponds to the primary immune effector phase. At this time point, DTH to soluble antigen was measured *in vivo* as an indicator of DTH reaction and CD4+ T cell activity. Moreover, the numbers of antigen specific IFN-γ producing CD8+ cytotoxic T cells were analyzed. Day 60 post-infection as well as day 5 post-challenge were chosen to analyze the memory immune effector phase [16]. To this end the number of memory effector T- cells was determined quantitatively.

**Figure 1:**
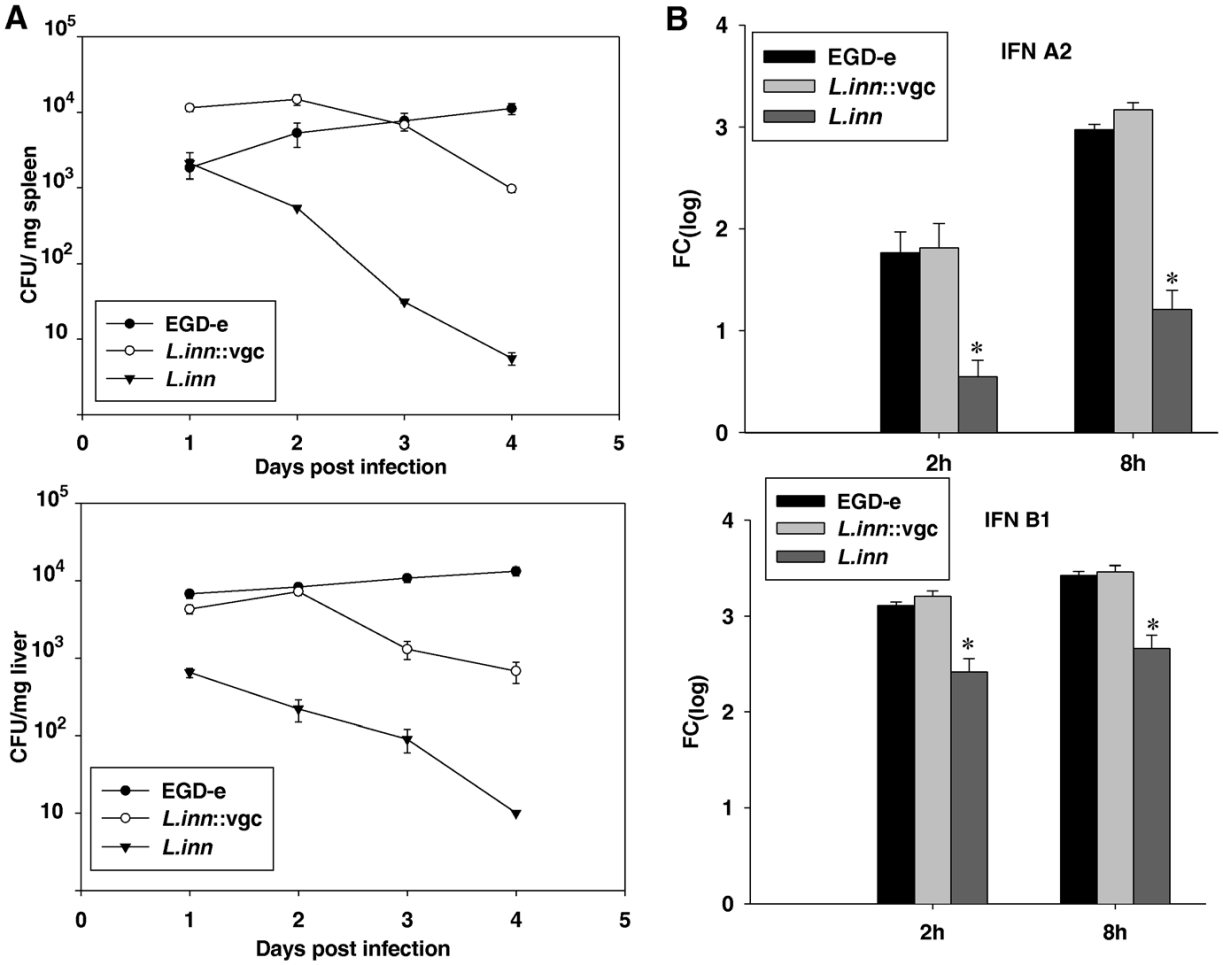
Bacterial load and IFN expression during the course of primary infection. **A.** Course of primary infection in mice with the wild type *Lm* (EGD-e) and the recombinant *L. inn*::vgc strain. Mice were infected i.v. with 10^3^ cfu *Lm*, 10^7^ cfu *L.inn*, or 10^7^ cfu *L.inn::vgc* strains. At different time intervals after the infection, mice were sacrificed and the number of viable bacteria in the organs was enumerated. **B.** Quantitative measurement of IFNα2 and IFNb1 expression in bone marrow-derived macrophages using RT-PCR at 2 h and 8 h following infection with *Lm* , *L.inn*, or the *L.inn*::*vgc* strains. *P<0.05 (*L.inn* vs. *Lm* and *L.inn::vgc* strains).

*In vivo* survival and growth kinetics of bacteria were followed by determining the number of bacteria in spleens and livers of infected mice. As expected, regardless of the dose of infection, the wild type *L.inn* strain was progressively cleared from both organs (Fig. 1A) whereas the *L.inn::vgc* strain successfully survived in both spleen and liver during the first two days after infection as indicated by the bacterial numbers that increased in both spleen and liver till day 2 and gradually decreased over days 3 and 4 post-infection. On the other hand, the bacterial numbers of the wild type *Lm*, increased from day 1 till day 4 post-infection in both spleen and liver.

### Stimulation of Type I interferon's by the *L.inn::vgc* strain

A striking phenomenon for cytosolic resident microbes is the ability to induce expression of Type I interferons. In contrast to the wild type *Lm*, its isogenic mutant lacking listeriolysin remains trapped in vacuoles and does not induce Type I interferon's [17]. We have recently documented that the *L.inn::vgc* can successfully survive inside phagocytic cells, thereby egressing from the phagolysosome [18]. In order to confirm if cytosolic persistence of the recombinant *L.inn::vgc* strain is efficient enough to stimulate production of such cytokines, we examined the transcriptional responses of IFN-a2 and IFN-b1 in bone marrow-derived macrophages following infection with *Lm*, *L.inn* as well as the recombinant *L.inn::vgc strain*. *L.inn::vgc* and the wild type *Lm* showed significantly higher transcriptional induction of both IFN-a2 and IFN-b1 than wild type *L.inn* at 2 hours post-infection (Fig. 1B). This effect was more pronounced at a later time point (8 hours) post-infection reflecting the efficient intracellular survival pattern of the *L.inn::vgc* strain.

### The recombinant *L.inn::vgc* strain exhibits a lowered inflammatory response

**Figure 2:**
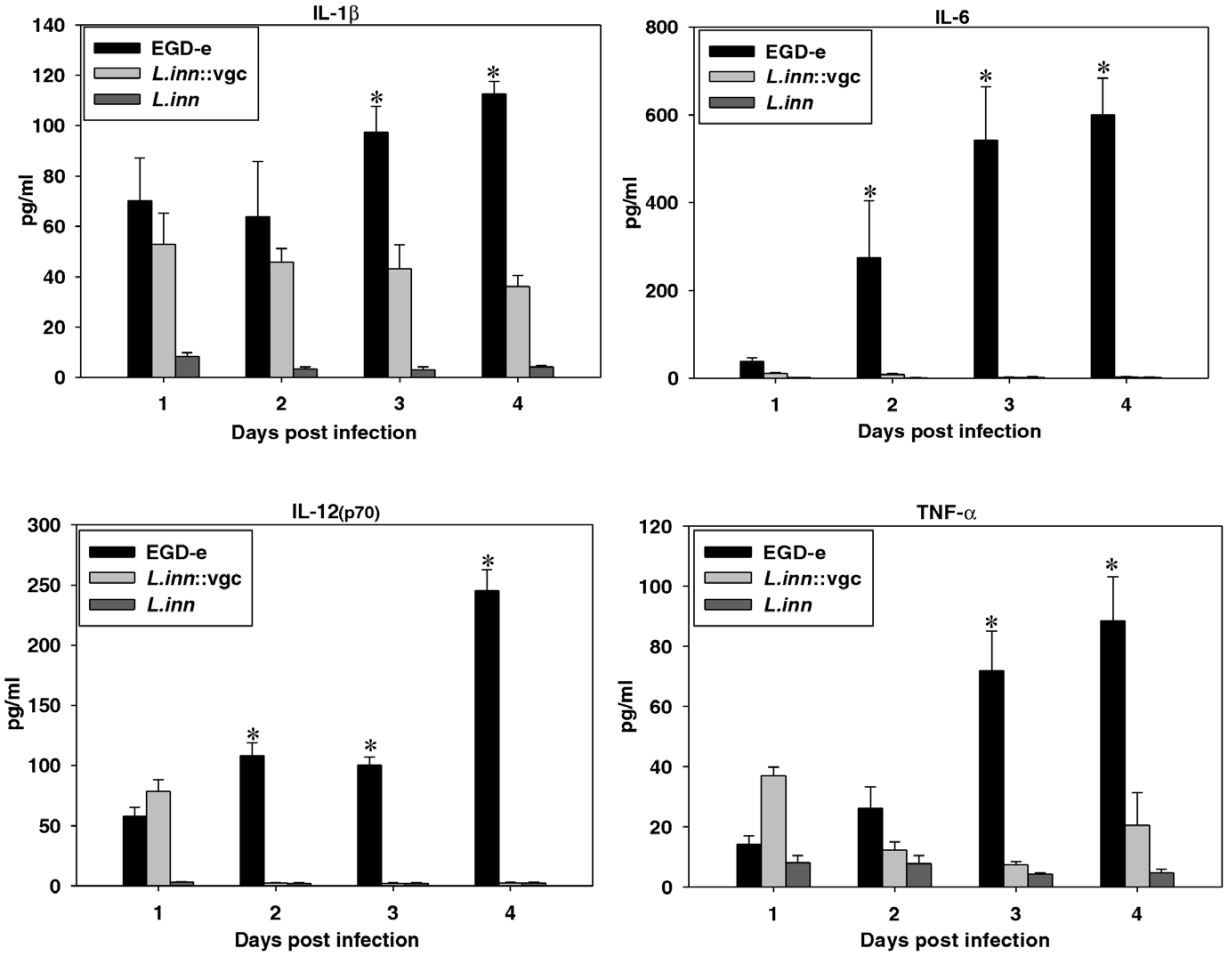
Measurement of proinflammatory cytokine levels in serum. Sera was obtained from mice on days 1, 2, 3, and 4 post-infection after inoculation with 10^3^ cfu *Lm*, 10^7^ cfu *L.inn*, or 10^7^ cfu *L.inn::vgc*. Levels of IL-1ß, IL-6, IL-12(p70), and TNF-alpha were quantified using a multiplex cytokine assay kit. *P<0.05 (EGD-e vs. *L.inn* and *L.inn::vgc* strains).

At the early stages of infection, wild type *Lm* is engulfed by professional phagocytes like macrophages, dendritic cells, or neutrophils. These cells produce a variety of proinflammatory cytokines which recruite or activate other inflammatory immune cells. The levels of IL-1ß, IL-6, IL-12, and TNF-alpha in mice sera were measured over the first 4 days after infection with *Lm* (10^3^), *L.inn* (10^7^), and the *L.inn::vgc* strain (10^7^). Like *L. inn,* , *L.inn::vgc* was not able to produce significant amounts of these cytokines over the first 4 days post-infection in spite of high infection doses (10^7^) while primary infection with *Lm* led to high proinflammatory cytokine production (Fig. 2).

**Figure 3:**
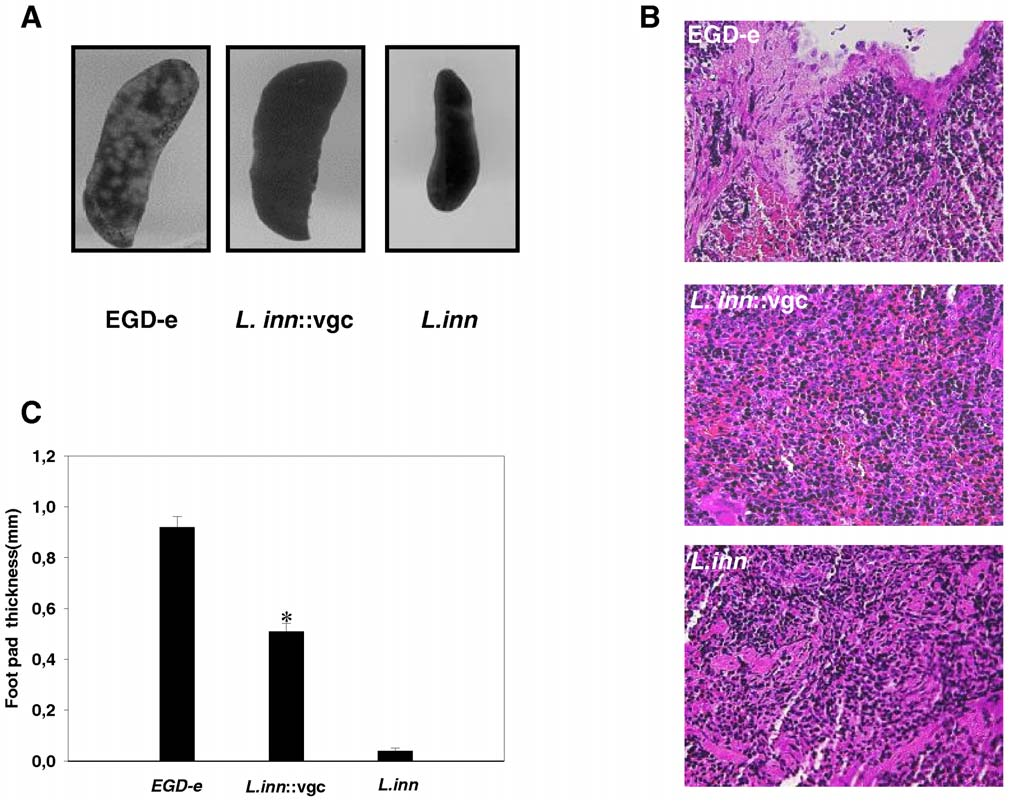
Examination of spleens and DTH response after infection with *Lm* and the recombinant *L.inn::vgc* strain. **A.** Morphological examination of spleens from mice inoculated i.v. with the wild type *Lm* and the recombinant *L.inn::vgc* strain. Spleens of mice infected i.v. as mentioned in Fig. 1 were isolated on day 3 after infection. Shown is a spleen from mice infected with the wild type *Lm*, the wild type *L.inn* and its recombinant mutant strain *L.inn::vgc*. Infiltration of monocytic cells and granulomatous lesions are only detectable in the spleens isolated from mice infected with the wild type *Lm*. **B.** Spleen sections were stained with HE and examined. Granulomas with massive leukocyte aggregates can only be detected in spleens of mice infected with *Lm*. **C.** DTH response to listerial antigen 9 days after primary infection. Mice were infected with 10^3^ CFU of *Lm*, 10^7^ CFU of *L.inn*, or 10^7^ CFU of *L.inn::vgc* strain. 9 days after infection, DTH was triggered through injection of soluble somatic listerial antigen. Twenty-four hours later, the specific skin response was determined. The mean value ± S.E. of five animals of a representative experiment is shown.*P<0.05 (EGD-e vs. *L.inn::vgc* strain).

Both granuloma formation and delayed-type-hypersensitivity footpad responses have previously been shown to be CD4^+^ T cell dependent inflammatory responses following infection with *Lm*. Morphological changes were examined in the spleens on day 3 after i.v. infection. Although the numbers of bacteria in spleens at day 3 post-infection for both *Lm* and *L.inn::vgc* were approximately the same (Fig. 1A), distinct differences in the morphological appearance between spleens isolated from mice infected with *Lm* and those isolated from mice infected with *L.inn::vgc* were observed (Fig. 3A). Splenomegaly associated with extensive granuloma formation was observed in spleens of *Lm* infected mice, as a result of intensive leukocyte infiltration which was visualized in stained spleen sections (Fig. 3B), whereas splenomegaly in the absence of granuloma formation was observed in spleens of *L.inn::vgc* infected mice. Infection with the wild type *L.inn* did not result in any morphological changes in spleens.

These observations were confirmed by antigen-elicited skin responses showing corresponding results (Fig. 3C). Mice were injected into the left hind footpads with 50 µl of somatic soluble *Lm* EGD-e antigen (60 ng/ml) at day 9 post-infection. Twenty-four hours later, thickness of the left and right footpads of individual mice were measured. Footpads of mice pre-immunized with *L.inn::vgc* showed reduced thickness than those of mice pre-immunized with the wild type *Lm*. The wild type *L.inn* strain did not induce a DTH response in the footpads of these mice. Moreover, antigen-induced CD4+ T cell-derived IFN-gamma production of spleen cells was measured as an indication for a pro-inflammatory T cell response. Spleen cells were isolated at day 9 post-infection and stimulated *in vitro* with the released soluble antigen of *L. monocytogenes* EGD-e (100 ng). Spleen cells from mice immunized with *L*.inn::vgc produced significantly lower levels of IFN-gamma when compared to spleen cells from mice immunized with wild type Lm. The wild type *L.inn* strain failed to prime T cells for the production of IFN-gamma (Fig. S1, supplementary information).

### Induction of T cell-mediated immunity by the recombinant *L.inn::vgc* strain

**Figure 4:**
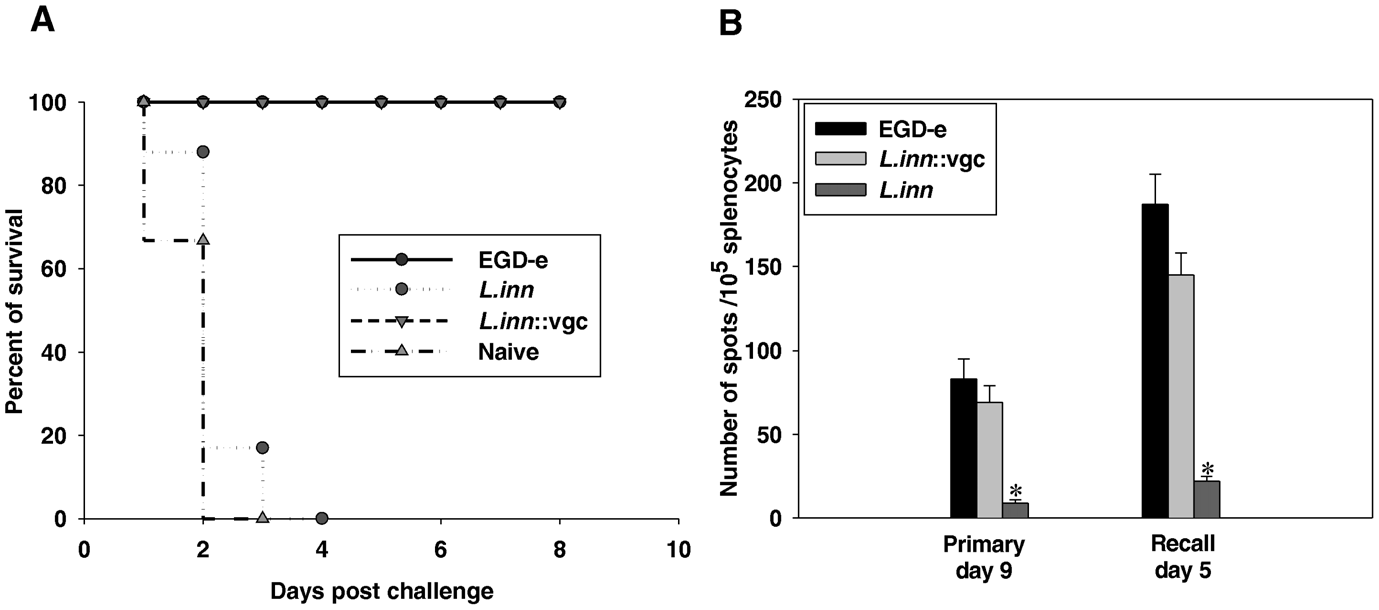
Protective immunity and cellular immune response after infection with *Lm* and the *L.inn::vgc* strain. **A.** Induction of protective immunity conferred after infection with the *L.inn::vgc strain*. Groups of 15 mice were infected i.v. as described in Fig. 1. Two months later all mice were challenged with a lethal dose (20×LD_50_) of the wild type *Lm*. As a control, a group of uninfected normal mice was included. Survival of mice after the challenge was monitored up to 8 days. **B.** Number of antigen-specific IFN-gamma producing CD8+ T cells in spleens of mice infected i.v. with the wild type *Lm*, *L.inn* and *L.inn::vgc* strain determined by ELISPOT. Spleen cells from infected mice were isolated either on day 9 after the primary infection or day 5 after challenge infection and stimulated with the immunodominant MHC class I peptide LLO_91–99_ in triplicates in nitrocellulose based 96-well culture plates. Number of specific IFN-gamma producing cells against the dominant H-2K^d^ restricted LLO_91–99_ epitope were determined by counting the number of spots under the microscope. *P<0.05 (*L.inn* vs. *Lm* and *L.inn::vgc* strains).

A number of cell types are involved in host defense against *Listeria*. Antigen-specific T lymphocytes mediate recovery from primary listerial infections and protective immunity to subsequent infections [13], [19]. Both CD4^+^ (helper, MHC class II restricted) and CD8^+^ (cytotoxic, MHC class I restricted) T cell subpopulations have been implicated [20]. Experimental evidence indicates, however, that CD8^+^ T cells play the predominant role in mediating protective immunity [21]–[24]. The ability of the recombinant *L.inn::vgc* to induce T-cell mediated immunity as a prerequisite for protective immunity was analyzed. Groups of BALB/c mice were infected with *Lm* (10^3^), *L.inn* (10^7^), or *L.inn::vgc* (10^7^). Two months later, all mice were challenged with a lethal i.v. dose (10^5^), corresponding to 20×LD50, of the wild type *Lm*, and survival was monitored. As controls, a group of untreated BALB/c mice that received a similar lethal dose of the wild type *Lm* were included. A single pre-immunization with the *L.inn::vgc* strain led to a significant protection against subsequent lethal infection with *Lm*. As expected, all mice that were pre-immunized with sub-lethal doses of *Lm* were also protected against a lethal listerial infection and survived whereas all non-immunized mice as well as those pre-immunized with *L.inn* died within 4 days after challenge (Fig. 4A).

Entry of *Listeria* into the cytosol is a critical event for CD8^+^ T cell recognition and induction of immunity [22]. In order to establish the correlation between the protection of mice pre-infected with *Lm* or the *L.inn::vgc strain* upon lethal challenge and the induction of CD8^+^ T cells in response to infection, the generation of antigen-specific MHC class I restricted CD8^+^ T cells were quantitatively examined. The numbers of antigen-specific MHC class I restricted effector CD8^+^ T cells induced in mice spleens 9 days after primary infection and 5 days after challenge with the wild type *Lm* (2×10^3^) was determined through evaluation of the number of IFN-γ producing CD8+ T cells induced showing reactivity against the dominant H-2K^d^ restricted LLO_91–99_ epitope [25] in an in vitro ELISPOT assay. As shown in Fig. 4B, infection with wild type *Lm* as well as the *L.inn::vgc* strain induced significant numbers of LLO_91–99_ specific CD8^+^ T-cells. After recall infection the numbers of LLO_91–99_ specific CD8^+^ T-cells showed a significant increase. On the other hand, infection with *L.inn* failed to induce a significant number of CD8^+^ T-cells either after primary infection or after challenge.

**Figure 5:**
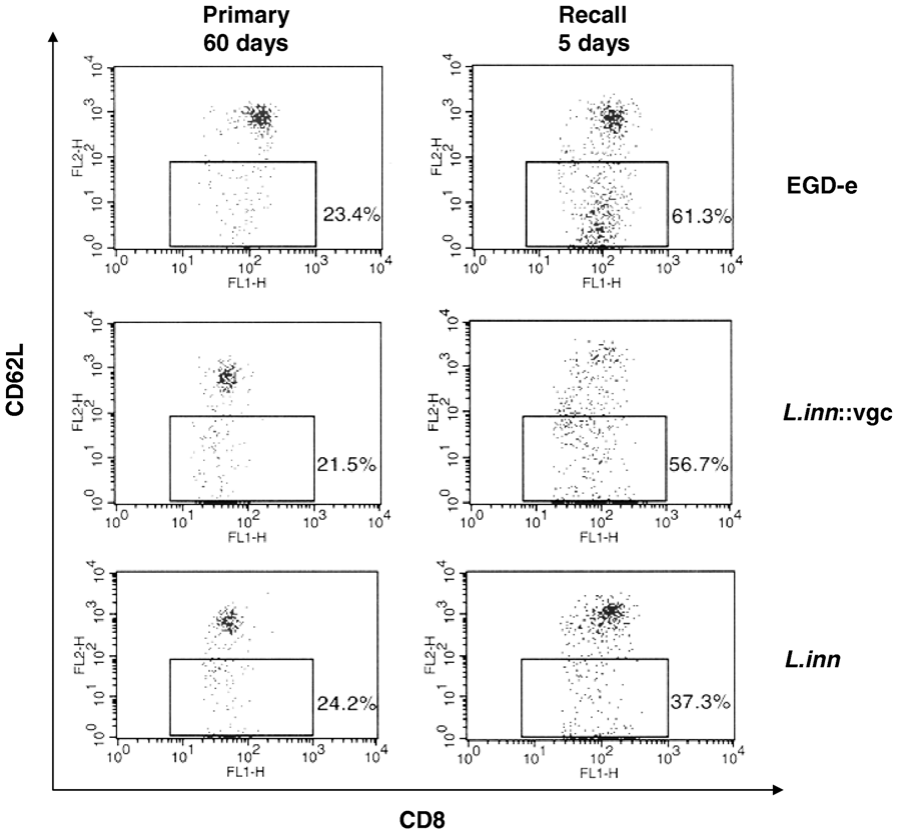
Expression levels of CD62L on CD8^+^ splenocytes following primary and recall infection with *Lm*, *L.inn* and the *L.inn::vgc* strain. Flow cytometry was performed on spleen cells, isolated from mice on day 60 after the primary infection or day 5 after the challenge. Cells were stained with FITC-labelled anti-Lyt-2 and biotinylated anti-CD62L. The binding of anti-CD62L on the cell surface was detected with PE-conjugated streptavidin. Numbers shown are gated CD8^+^CD62^lo^ T cells and analyzed with CELLQuest software.

To address the contribution of effector memory CD8+ T cells in mediating long-lasting immunity after re-infection with the wild type *Lm*, the expression level of the cell surface adhesion molecule CD62L was quantified. Expression of CD62L is down regulated on the surface of cytotoxic CD8^+^ T-cells when developed to protective memory T cells [21]. Two months after the primary infection, the number of CD8^+^CD62L^lo^ lymphocytes was approximately identical in all groups of primarily infected mice. This number increased dramatically upon re-infection with the wild type *Lm* (2×10^3^) in mice pre-immunized with *Lm* as well as with *L.inn::vgc* while pre-immunization with *L.inn* was not able to induce CD62L down-regulation seen in the other groups (Fig. 5). In addition we have monitored the expression of CD44 on CD8^+^ T-cells. CD44 is expressed at high levels on memory but not in naïve T-cells [26]. In mice that were primarily infected with *Lm* and *L.inn::vgc*, the expression of CD44 was upregulated on CD8^+^ T-cells 5 days post-challenge infection with the wild type *Lm* (2×10^3^) while primary infection with *L.inn* did not lead to a significant change in CD44 expression pattern (Fig. S3).

## Discussion

In this study, we define a unique vaccine strategy which is based on a rationally designed pathogen by complementation of a non-pathogenic strain with selected genes necessary to induce a vigorous immune response. To illustrate the proof of principle of this strategy we used the *Listeria* model by taking a non-pathogenic *L.inn* strain and complementing it with genes from pathogenic *Lm* which were previously shown to be a *sine qua non* requirement for intracellular growth and survival [18]. This novel vaccine strategy resulted in generation of a recombinant strain (*L.inn::vgc*) that possesses properties needed to induce the marked protective immunogenic properties of the wild type *Lm* but is attenuated in virulence as well as in its capacity to induce host detrimental cell-mediated inflammation. The recombinant *L.inn::vgc* strain showed a significant *in vivo* survival rate in the first 3 days post-infection (Fig. 1A). This observation is in accordance with our recent finding that the *L.inn::vgc strain* is able to survive in phagocytic host cells [18]. Moreover, it has the capability to induce identical Type I interferon at levels similar to wild type *Lm*. As previously shown by McAffrey *et al*., 2003, induction of type 1 IFN is a surrogate marker indicating access of Listeria into the cytosol of antigen presenting cells [17]. We have shown that the *L.inn::vgc strain* could use the complemented virulence factors to escape into the cytosol and subsequently be presented to CD8 T lymphocytes. In this context Zwaferink et al. 2008 have shown a role for IFNb in macrophage cell death. Treatment of macrophages with this cytokine could enhance host-cell membrane permeabilization by listeriolysin consequently leading to cell apoptosis [27].

Especially encouraging was the observation that, although *L.inn::vgc* strain was injected at a high dose of 10^7^ cfu, mice could still efficiently control the infection and showed very low blood levels of pro-inflammatory cytokines thus reducing the detrimental inflammatory responses caused by the wild type *Lm*. Morphological and histological analysis of spleen after infection with *Lm* have shown the induction of splenomegaly and granuloma as a result of monocytic infiltrations of the white pulp which were most pronounced on day 3 post-infection while infection with *L.inn::vgc* only resulted in a splenomegaly without any significant morphological changes detectable (Fig. 3A,3B). The intensity of the morphological and histological alterations in spleens paralleled the level of *Listeria*-induced DTH responses, as the *in vivo* induction of DTH after *L.inn::vgc* infection was also significantly lower than DTH induction following *Lm* infection (Fig. 3 and Fig. S1). We therefore show that the recombinant *L.inn::vgc* strain shows a significantly reduced proinflammatory and CD4^+^ mediated inflammatory response compared to *Lm*, thereby addressing one major concern regarding the use of live *Lm* as a vaccine.

However the crucial question remained as to whether the *L.inn::vgc* strain elicits significant adaptive immune responses resembling those of the wild-type strain. Since *Lm* is located in both phagosomes and cytosol of professional antigen-presenting cells during infection, epitopes derived from *Listeria* proteins are presented by the MHC pathway thereby priming both effector CD4^+^ and CD8^+^ T cells [28], [29] resulting in full elimination of *Listeria* from the host. Previous experimental studies [30], [31] have revealed that persistence and number of viable microorganisms are important parameters for establishing efficient T cell-mediated immunity. Moreover, it has been shown that the presence of live bacteria in mice organs over the first 48 hours after immunization is critical for the induction of effector CD8^+^ T cell mechanisms [32]. Indeed we were able to show that all animals immunized with *Lm* or *L.inn::vgc* were protected against 20×LD_50_ of virulent *Listeria* (Fig. 4A). Although the *L.inn::vgc* strain elicits lowered CD4^+^ mediated inflammatory responses as compared to infection with *Lm*, it is capable of mounting a successful anti-*listerial* protective response, indicating that the observed *in vivo* survival pattern of the *L.inn::vgc* strain was sufficient to induce protection.

The entry of effector T cells into a memory stage, however, is accompanied by the ability to rapidly expand their population during recall responses and to down regulate expression of cell surface markers such as CD62L and CCR7 [33]. It was previously reported that primary infection with the wild type *Lm* induces down regulation of CD62L on the surface of effector CD8^+^ T cells which reaches its lowest levels at day 8 post-infection [34]. However, over the following weeks, expression of CD62L is up regulated. During recall infection, CD62L is then rapidly down regulated on the surface of memory CD8^+^ T cells [32], [35]. In order to correlate protection against challenge with *Listeria* with antigen specific CD8^+^ T cells, we examined the induction of LLO_91–99_ specific CD8^+^ T cells in response to primary infection with the different *Listeria* strains. Infection with *L.inn::vgc* induced a significant population of cytotoxic CD8^+^ T lymphocytes (Fig. 4B) which, upon challenge with the wild type *Lm*, showed a CD62L expression pattern similar to that presented in mice primarily infected with the wild type *Lm* (Fig. 5). The identity of the memory T-cells induced in response to *L.inn::vgc* infection was confirmed by testing the CD44 expression on the CD8^+^ T-cells following recall infection with *Lm* where high expression of CD44 was observed on CD8^+^ T-cells derived from mice primarily infected with the recombinant *L.inn::vgc* strain but not with the wild type *L.inn* (Fig. S3). The inability of the *Lm* strain lacking listeriolysin O (LLO) [36] as well as the *L.inn* strain expressing only LLO [37] to induce a protective T cell response reflects the requirement of the entire virulence gene cluster in conferring a long lasting immunity. We therefore show that a non-pathogenic *L.inn* strain complemented with the entire *vgc* is capable of inducing a vigorous anti-*listerial* response.

Even though we have demonstrated a vigorous immune response following i.v. infection the immune response to Listeria can vary considerably depending on the route of administration. Using the intraperitoneal route of infection, we obtained a similar result i.e. protection following pre-infection with the *Lm* and *L. inn::vgc* strains but not with mice pre-immunized with the *L. inn* strain (Fig. S2). Thus despite a different route of infection *Lm::vgc* is able to induce protection in-vivo. The mouse is not a suitable and reproducible model for evaluating oral immunization protocols because of the specificity of the listerial InlA molecule [38]. Therefore experiments examining mucosal immunity will have to be carried out in the guinea pig model of listerial infection.

Recently, highly attenuated mutants of *Lm* have been developed as candidates for vaccine vectors [3], [39], however, a single immunization with these strains was not sufficient for the induction of protective cellular immunity.

Here a transcomplemented strain of a non-pathogenic *L. inn* strain expressing genes of the *vgc* cluster provides robust protection with a single dose of 10^7^ cfu bacteria without causing any signs of overt illness. The LD50 of the wild type Lm is around 5000 cfu. As shown in figure 1A, the *L.inn::vgc* strain does not grow *in-vivo* beyond day 3 post-infection and is subsequently eliminated. These properties, imparting protective responses and rapid elimination from the host are considered to be desirable properties for successful vaccine vectors. Our results, namely, the *in vivo* survival pattern, the induction of interferon's and antigen specific CD8^+^ T cells, the lack of overt detrimental inflammatory reactions and most importantly the induction of protection against challenge with *Listeria*, allow the conclusion that the *L.inn::vgc strain* is potentially capable of inducing protection and that further development of this strain as a suitable live bacterial vaccine vector in clinical settings are warranted.

## Materials and Methods

### Ethics Statement

Mice experiments were done according to the requirements of Justus-Liebig University Giessen Animal Ethics Committees with ethics approval number: 63/2007. Animals were sacrificed using CO2 asphyxiation and the appropriate organs aseptically harvested.

### Mice

Six to eight week-old female BALB/c mice, purchased from Harlan Winkelmann (Borchen, Germany), were kept at our breeding facilities in specific-pathogen-free conditions and used in all experiments.

### Bacteria

Bacterial strains used in this study are wild type *Listeria monocytogenes* EGDe serotype 1/2a (*Lm*) [40], wild type *L. innocua* strain (serotype 6a NCTC 11288) [41] transformed with either the recently characterized gram+ve/gram-ve shuttle pUvBBAC+*vgc*1 vector and referred to as (*L.inn::vgc* strain) or the pUvBBAC vector without the inserted *vgc* and referred to as *L.inn* [18] Bacteria were grown in brain-heart infusion (BHI) (Difco, Augsburg; Germany) broth in presence or absence of 5 µg/ml erythromycin. For each experiment, erythromycin was used as a selective antibiotic for growth of *L.inn::vgc* and the wild type *L.inn* harbouring the pUvBBAC vector. Wild type L. monocytogenes was grown in absence of erythromycin. In all experiments, fresh cultures of bacteria, prepared from an overnight culture, were used. Briefly, bacteria were grown in Brain Heart Infusion (BHI) at 37°C, harvested in the exponential growth phase and washed twice with PBS. The pellet was resuspended in PBS and the bacterial concentration was calibrated by optical absorption. Further dilutions were prepared in PBS to obtain required numbers of bacteria for infection.

### *In vitro* infection assay

The protocols for animal handling were previously approved by our institutional Animal Ethics Committee (protocol number 63/2007). Bone marrow-derived macrophages were isolated from 4 to 6 week old C57Bl/6 female mice and grown and differentiated for 7 days in L929 conditioned medium to an approximate concentration of 2,5×10^5^ cells/well in 6-well plates. On the day of infection the medium was exchanged against MDEM medium with 1% FCS and the cells were infected with 5×10^6^ cfu per well with the wild type *Lm* and *L.inn* strains as well as the recombinant *L.inn::vgc* strain for 2 h and 8 h. The cells were lysed and their total RNA was isolated.

### RNA isolation

For every bacterial strain and negative control the cells of at least two wells of a six well tissue culture plaque were lysed and total RNA was isolated. Prior to lysis culture medium was aspirated and cells were lysed using RLT lysis buffer (Qiagen, Germany). Total RNA was isolated using the RNeasy Mini Kit and the RNase free DNase I set (Qiagen) following the manufacturers protocol. The RNA was recovered in RNase free water, heat denatured for 10 min. at 65°C; quantified with the NanoDrop® ND-1000 UV-Vis Spectrophotometer (NanoDrop Technologies, USA) and a quality profile with the Agilent 2100 bioanalyzer (Agilent Technologies, Germany) was made.

### Real time RT-PCR

First-strand cDNA was synthesized with 500 ng of purified RNA using SuperScriptII (Invitrogen) and a mixture of T21 and random nonamer primers (Metabion) following the instructions for the reverse transcription reaction recommended for the QuantiTect SYBR Green PCR Kit (Qiagen). Real-time quantitative PCR was performed on an ABI Prism 7700 real time cycler. The relative expression of the targets IFNa2 (Interferon alpha 2) and IFNb1 (Interferon beta) were normalized to that of two reference genes: SDHA (Succinate dehydrogenase alpha subunit) and PPIA (peptidylprolyl isomerase A). Finally a mean of the fold change of the target versus each of the reference genes was taken as the final value.

### Somatic bacterial antigens

Somatic soluble antigen was prepared by culturing *Lm* in tryptic soy broth for 18 h, washing it in PBS, and subsequently subjecting it to ultrasonication.1 g (wet weight) of bacterial cells were suspended in 10 ml of PBS and sonicated five times for 1 min (87.5%, output, degree 7 on a sonifier model S-125; Branson Sonic Power, USA) on ice. The sonicated suspension was centrifuged at 39 000 U for 50 min, and the supernatant was filter sterilized (pore size,0.45 µm) and stored at −20°C at a dilution of 1∶100 in PBS [42].

### Experimental infection and determination of bacterial load in infected organs

Primary *in vivo* infection with *Lm* (10^3^), the wild type *L.inn* (10^7^), or the *L.inn::vgc* (10^7^) strain was performed by an intravenous injection of viable bacteria in a volume of 0.2 ml PBS. Bacterial growth in spleens and livers was determined by plating 10-fold serial dilutions of the organ homogenates on BHI agar plates. The detection limit of this procedure was 10^2^ colony forming units (CFU) per organ. Colonies were counted after 24 h of incubation at 37°C.

### Measurement of cytokines production

Cytokine production was assayed from the collected sera of infected mice using a multiplex cytokine assay kit and Luminex technology (Bio-Rad). Balb/C mice were infected with *Lm* (10^3^), the wild type *L.inn* (10^7^), or the *L.inn::vgc* (10^7^) strain. Sera were aseptically isolated on days 1, 2, 3, and 4 post-infection. Four cytokines were tested: TNFα, IL-1β, IL-6, and IL-12(p70) and cytokine levels were presented as absolute concentrations in pg/ml.

### Histology

Spleens were aseptically isolated from mice previously infected with the different *Listeria* strains as mentioned above and examined for morphological alterations. Tissues were fixed in 10% neutral buffered formalin, embedded in paraffin, sectioned, and 5 mm sections were stained with hematoxylin and eosin (HE). Pathoplogical foci in spleen sections were then microscopically examined (Keyence).

### Estimation of antigen specific IFN-γ producing cells

Spleens were aseptically removed from mice at day 9 post-infection with the wild type *Lm*, the wild type *L.inn*, or the *L.inn::vgc* strain. Spleen cells were isolated and antigen (LLO_91–99_) specific IFN-γ producing CD8+T cells were determined in the spleens of mice after i.v. infection with the same bacterial strains mentioned above by using an ELISPOT system as previously described [23], [35].

### Quantification of IFN-gamma in cell culture supernatants

IFN-gamma was measured in the supernatants of splenocytes by using a mouse IFN-gamma ELISA kit, BD optEIA™ (BD Biosciences Pharmigen) according to the manufacturer instructions. The assay was performed in duplicates, and data represent means ± standard error.

### Flow cytometry analysis

For flow cytometry analysis, approximately 1×10^6^ splenocytes, isolated from infected mice (*Lm*, *L.inn*, and *L.inn::vgc* strains) were stained with FITC labelled anti-CD8 and biotinylated anti-CD62L or anti-CD44 (pharMingen, Becton Dickinson). PE-conjugated streptavidin was used to detect the binding of anti-CD62L or anti-CD44 on the cell surface. Flow cytometry was performed using a FACS Calibur flow cytometer and further analyzed with CELL Quest software (Becton Dickinson, CA).

### Protection studies

All mice, pre-immunized with wild type *Lm*, the wild type *L.inn* strain and the *L.inn::vgc* strain were challenged 2 months later with a 20×LD_50_ (10^5^) lethal dose of wild type *Lm*. A group of non pre-immunized Balb/c mice were included as controls. Survival of mice was monitored for several days and expressed as percentage of animals surviving challenge with *Lm*.

### Statistical analysis

Data are representative of at least three independent experiments. Significance of the represented data was calculated using ANOVA (analysis of variance). Data are expressed as mean ± standard errors (S.E.).

## Supporting Information

Figure S1 ***Listeria*** **-induced IFN-gamma production by spleen cells 9 days after infection (i.v.).** Mice were infected with 10^3^ CFU of *Lm*, 10^7^ CFU wild type *L.inn*, or with 10^7^ CFU of *L.inn*::vgc strain. On day 9 after infection, mice were killed and spleens removed. Single cell suspensions were stimulated *in vitro* with secreted soluble *Listeria* antigen to produce IFN-gamma. After 48 hours, culture supernatants were tested for presence of IFN-gamma by ELISA. *P<0.05 (EGD-e vs *L.inn*::vgc).
(TIF)

Figure S2 **Intraperitoneal infection with the** ***L.inn*** **::vgc strain induces protective immunity.** Mice were infected intraperitoneally with *Lm*, *L.inn* and the *L.inn::vgc* strain as described in figure 4A. After 2 months all mice were challenged i.v. with a lethal dose (20×LD_50_) of the wild type *Lm*. As a control, a group of uninfected normal mice was included. Survival was monitored up to 8 days after challenge.
(TIF)

Figure S3 **Quantification of CD44 expression on CD8^+^ splenocytes following primary and recall infection with** ***Lm*** **,** ***L.inn*** **and the** ***L.inn::vgc*** **strain.** Flow cytometry was performed on spleen cells, isolated from mice on day 60 after the primary infection or day 5 after the challenge. Cells were stained with FITC-labelled anti-Lyt-2 and PE-labelled anti-CD44. Numbers shown are gated CD8^+^CD44^hi^ T cells and analyzed with CELLQuest software.
(TIF)
