# Supplementary figures and images for: Protective Immunity to Listeria Monocytogenes Infection Mediated by Recombinant Listeria innocua Harboring the VGC Locus
Source: PLoS One. 2012 Apr 19;7(4):e35503. doi: 10.1371/journal.pone.0035503 (PMC3334901; doi:10.1371/journal.pone.0035503)

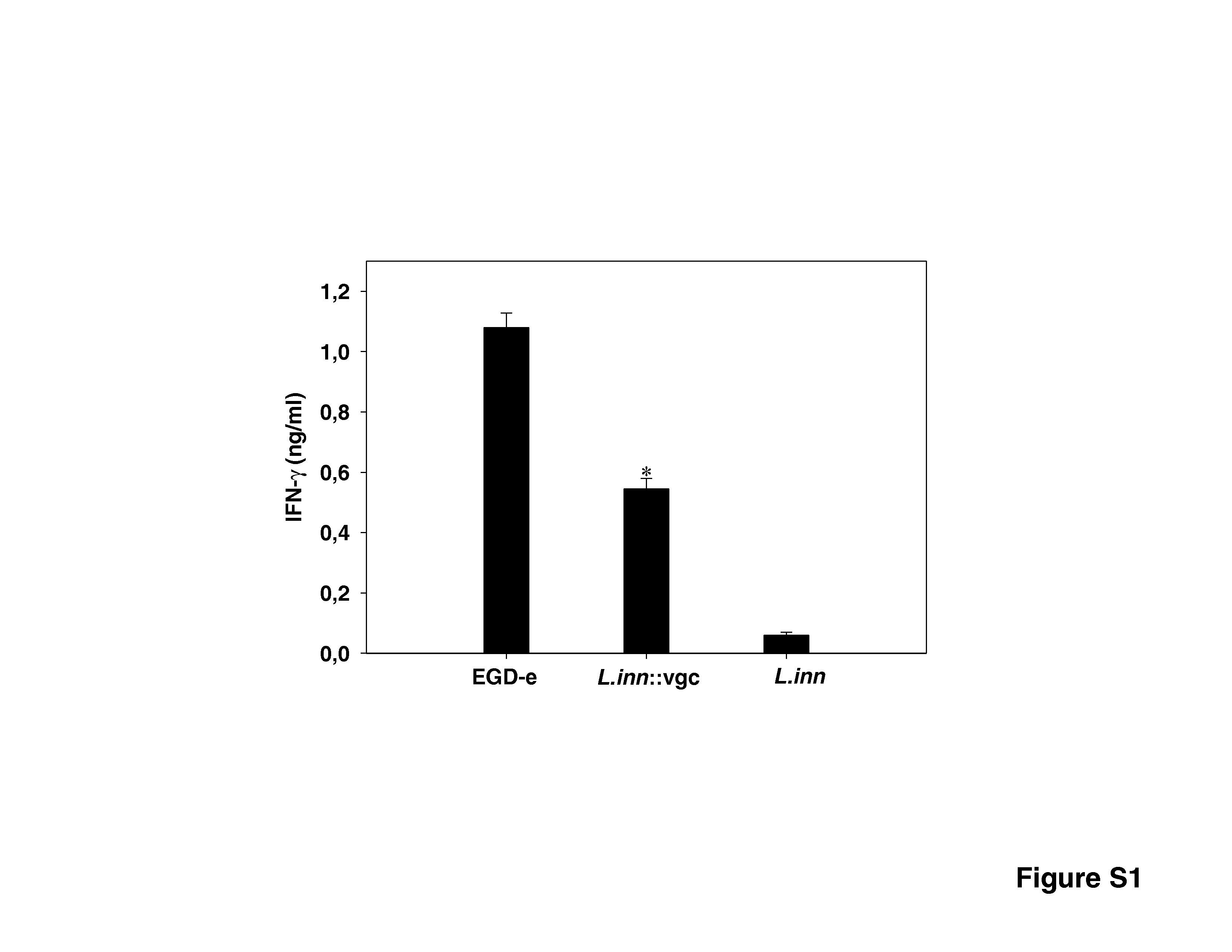

Supplement: Figure S1 — Listeria -induced IFN-gamma production by spleen cells 9 days after infection (i.v.). Mice were infected with 103 CFU of Lm, 107 CFU wild type L.inn, or with 107 CFU of L.inn::vgc strain. On day 9 after infection, mice were killed and spleens removed. Single cell suspensions were stimulated in vitro with secreted soluble Listeria antigen to produce IFN-gamma. After 48 hours, culture supernatants were tested for presence of IFN-gamma by ELISA. *P<0.05 (EGD-e vs L.inn::vgc). (TIF) [file pone.0035503.s001.tif]

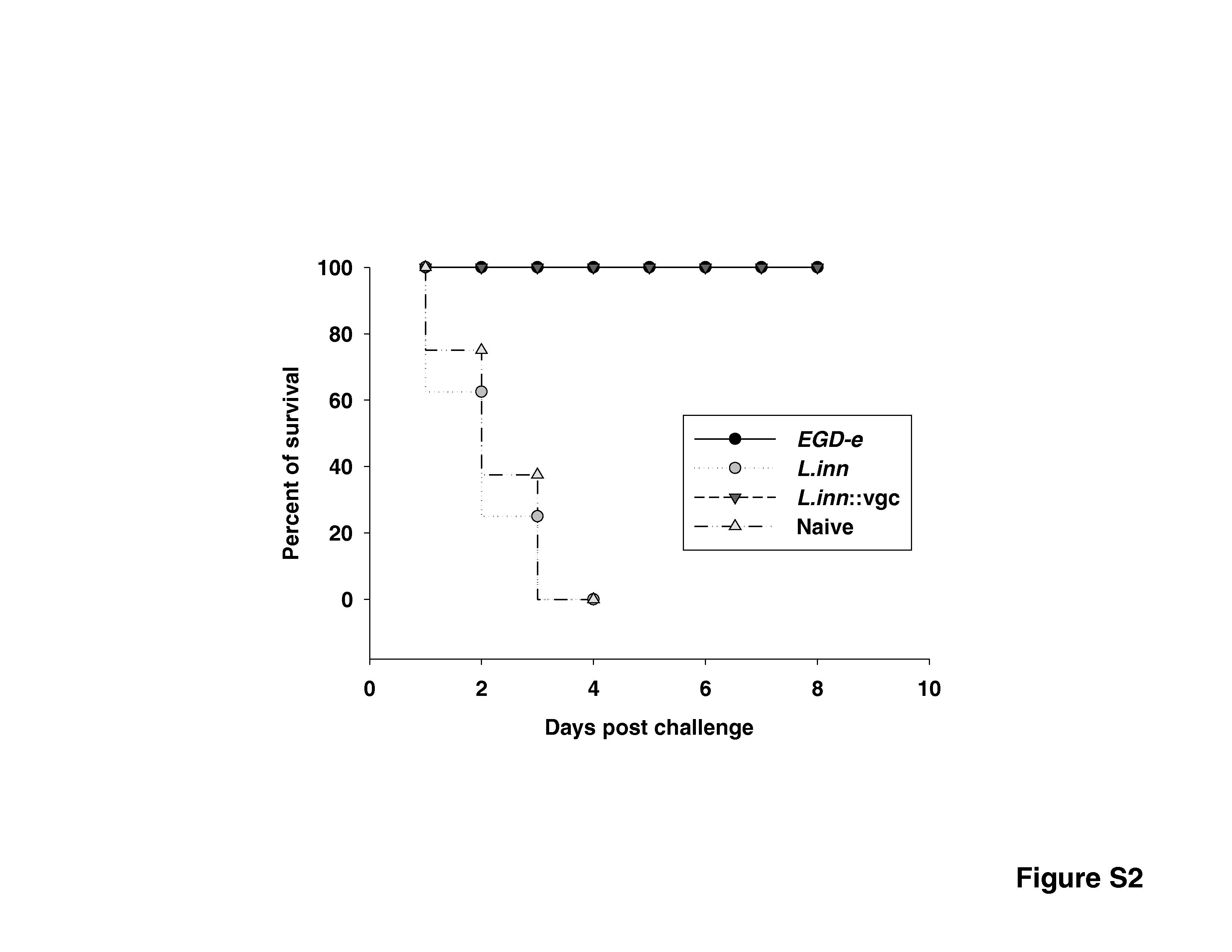

Supplement: Figure S2 — Intraperitoneal infection with the L.inn ::vgc strain induces protective immunity. Mice were infected intraperitoneally with Lm, L.inn and the L.inn::vgc strain as described in figure 4A. After 2 months all mice were challenged i.v. with a lethal dose (20×LD50) of the wild type Lm. As a control, a group of uninfected normal mice was included. Survival was monitored up to 8 days after challenge. (TIF) [file pone.0035503.s002.tif]

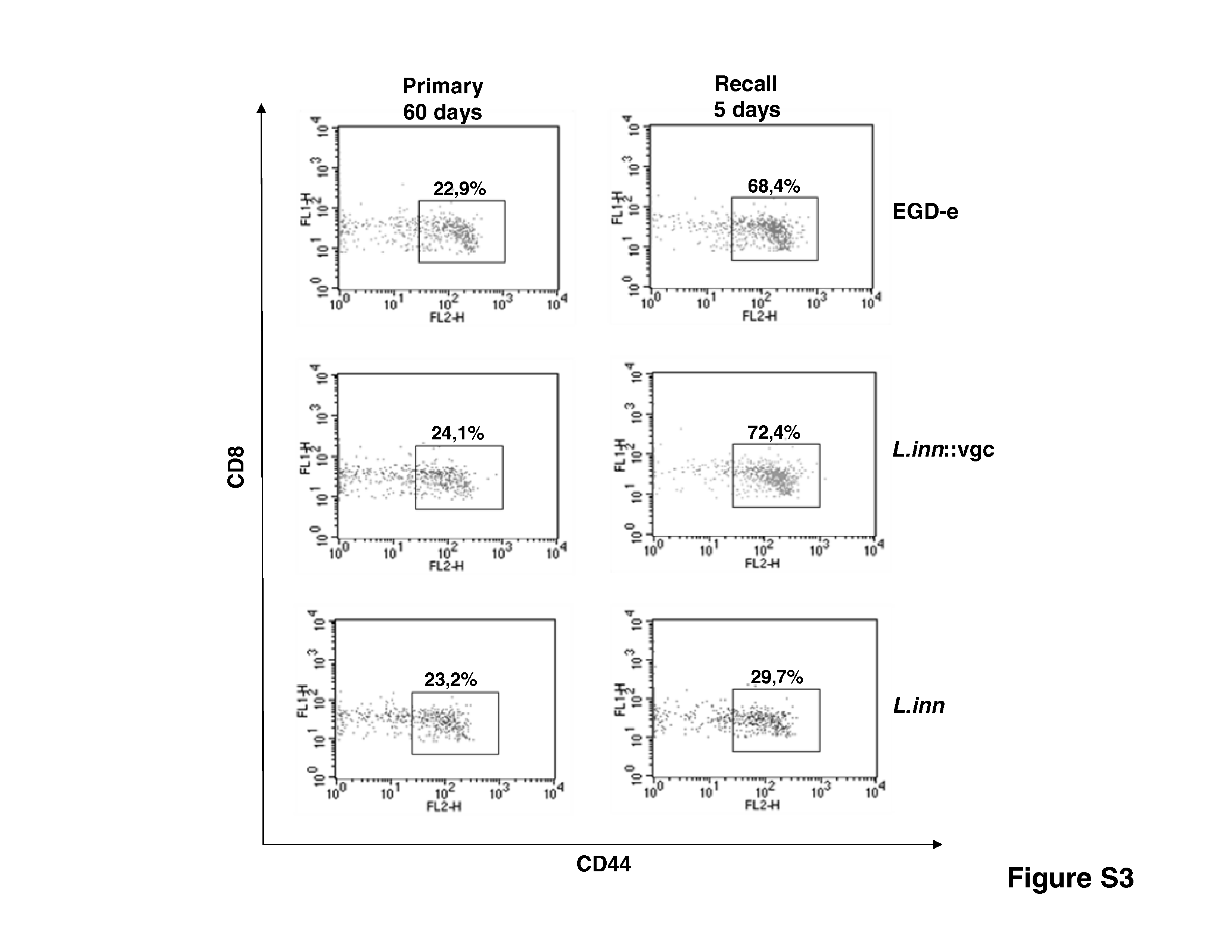

Supplement: Figure S3 — Quantification of CD44 expression on CD8+ splenocytes following primary and recall infection with Lm , L.inn and the L.inn::vgc strain. Flow cytometry was performed on spleen cells, isolated from mice on day 60 after the primary infection or day 5 after the challenge. Cells were stained with FITC-labelled anti-Lyt-2 and PE-labelled anti-CD44. Numbers shown are gated CD8+CD44hi T cells and analyzed with CELLQuest software. (TIF) [file pone.0035503.s003.tif]
